# An Analog Sensor Signal Processing Method Susceptible to Anthropogenic Noise Based on Improved Adaptive Singular Spectrum Analysis

**DOI:** 10.3390/s25051598

**Published:** 2025-03-05

**Authors:** Zhengyang Gao, Shuangchao Ge, Jie Li, Wentao Huang, Kaiqiang Feng, Chenming Zhang, Chunxing Zhang, Jiaxin Sun

**Affiliations:** 1 National Key Laboratory for Electronic Measurement Technology, North University of China, Taiyuan 030051, China; livingwinds@163.com (Z.G.); lijie@nuc.edu.cn (J.L.); fkq0809@163.com (K.F.); s202206050@st.edu.cn (C.Z.); sunjx621@163.com (J.S.); 2 Taiyuan Satellite Launch Center TSLC, Taiyuan 030027, China; hwt.234@163.com; 3 State Key Laboratory of Geodesy and Earth’s Dynamics, Innovation Academy for Precision Measurement Science and Technology, Chinese Academy of Sciences, Wuhan 430077, China; zhangchenming24@mails.ucas.ac.cn

**Keywords:** singular spectrum decomposition, data acquisition, data processing, parameter estimation

## Abstract

Sensor measurements are often affected by complex ambient noise and complicating signal processing tasks. The singular spectrum decomposition (SSA) algorithm, while widely used, faces challenges such as the difficulty of determining the number of decomposition layers, requiring iterative adjustments that reduce precision and increase processing time. This paper proposes an improved adaptive singular spectrum analysis (ASSA) algorithm that integrates a deep residual network (Res-Net) for automatic recognition. A comprehensive interference signal database was constructed to train the Deep Res-Net, and common interferences were restored through the combination of different signals, enabling greater frequency resolution performance. Meanwhile, a novel correlation detection reconstruction method based on a clustering algorithm for adaptive signal classification was developed to suppress background noise and extract meaningful signals. ASSA addresses the challenge of determining the optimal number of decomposition layers, eliminating the parameter adjusting process and enhancing the measurement efficiency of sensor systems. Through experiments, magnetotelluric (MT) observation data with complex interferences were applied to demonstrate the performance of ASSA, and promising results with an RMSE of 0.2 were obtained. The experiments also showed that the accuracy of ASSA was improved by 14% compared to other signal extraction algorithms, proving that ASSA can achieve excellent results when applied to other data processing fields.

## 1. Introduction

In the field of sensor measurement, analog signal sensors have important and wide application scenarios [1]. However, in the actual measurement process, the sensor signal can be weak and it is very susceptible to human interferences, such as power line interference, switch interference, drift current interference and vehicle noise etc. [2]. Normally, as a kind of non-linear and non-stationary signal, the analog sensor data cannot be well processed using methods based on linear and stationary assumptions [3,4].

In the realm of signal processing, the endeavor to mitigate noise interference and extract pertinent components has emerged as a research hotspot. In recent years, singular spectrum analysis (SSA) has gained attention as an effective method for dealing with nonlinear signals [5] due to its nonparametric and nonstationary assumptions [6]. Nevertheless, traditional SSA encounters challenges in accurately discerning useful components within intricate signals, as it struggles to distinguish between the singular values of useful and noisy signals. This paper provides an improved adaptive singular spectrum analysis (ASSA) algorithm to improve the effectiveness of signal recognition and extraction, especially when processing signals embedded in complex background noise.

Conventional techniques for processing non-stationary signals predominantly rely on mathematical and statistical methodologies. Researchers have explored a plethora of ways to address this daunting challenge, including variational mode decomposition [7], adaptive chirp mode decomposition and neural networks [8]. In the quest to efficiently mitigate interferences, early-stage efforts often resort to the least squares method for impedance estimation in MT analysis [9], which proves effective in attenuating Gaussian noise [10,11]. Sharma et al. introduced SSA into automatic recognition of electrocardiogram (ECG) measured signals [12]. Wang et al. combined multichannel SSA with affinity propagation to reduce the interference of ranging sensors [13]. The above methods all have certain limitations, can only guarantee a good effect in a certain signal interval, and cannot achieve satisfactory results in global signal processing [14].

Singular spectrum analysis (SSA) is a powerful technique for global time series analysis [15], which is widely applied in the realms of time series analysis [16], classification problems [17], fault recognition [18,19] and non-stationary signal decomposition [20]. Based on the idea of phase space reconstruction, SSA can extract different components of a complex signal using singular value decomposition (SVD) [21]. It can effectively separate nonstationary signals with large temporal differences when the length of the orbital matrix and the number of decomposition layers are sated. In 2017, Armouche et al. combined SSA with an unsupervised classification algorithm and proposed sliding SSA [22], which can effectively extract two sinusoidal signals with similar frequencies. Zhang et al. introduced SSA to extract feature signal extraction when dealing with electric current magnetic interference in magnetic sensors [23]. Lin et al. employed SSA to estimate the modal parameters of engineering structural systems [24].

However, deficient or excessive decomposition will occur under improper prior and posterior parameters for SSA. Singular spectrum decomposition (SSD) is a new adaptive method for decomposing non-linear and non-stationary time series in narrow-banded components [25]. SSD was developed based on SSA, which can adaptively select the embedding dimension and decompose the original signal into several singular spectral components from high frequency to low frequency. Weiyang et al. utilized improved singular spectrum decomposition (SSD) and a singular-value energy autocorrelation coefficient spectrum to extract the rolling bearing fault feature [26]. SSD is highly adaptable, but it cannot decompose similar components [27]. The singular values of the above method still need to be manually adjusted, the time spent on adjusting cannot be quantified, and the results may achieve sub-optimal values. In addition, the resolution performance of similar frequencies of the above methods still needs to be improved.

To effectively decompose interference and preserve critical MT components, this paper proposes an enhanced SSA method incorporating automatic aggregation classification. A comprehensive noise database, encompassing typical MT noise types, is constructed to train a Deep Res-Net, endowing the model with superior frequency resolution capabilities. Building on this foundation, an intelligent signal classification and recognition framework is developed using Deep Res-Net to accurately identify analog sensor signals which are suspectable to human interferences. The exceptional frequency resolution of Deep Res-Net significantly enhances the algorithm’s overall adaptability. Furthermore, a novel correlation detection signal reconstruction method based on K-means clustering is introduced. By employing a correlation matrix model to constrain clustering, this approach eliminates manual parameter adjusting, ensuring globally optimal extraction results and improving the accuracy of target signal extraction. Simulation experiments demonstrate that ASSA achieves superior frequency resolution, with a more than 14% increase in accuracy compared to conventional signal extraction algorithms. Validation with MT observation data reveals that ASSA achieves a root mean square error (RMSE) of 0.2, demonstrating its high precision in processing analog sensor signals.

The rest of this paper is organized as follows: the second part introduces the method, the third part describes the experiment and data analysis, and finally, the conclusion is given.

## 2. Methodology

### 2.1. Basic Theory of Singular Spectrum Analysis and K-Means Clustering Algorithm

**Figure 1:**
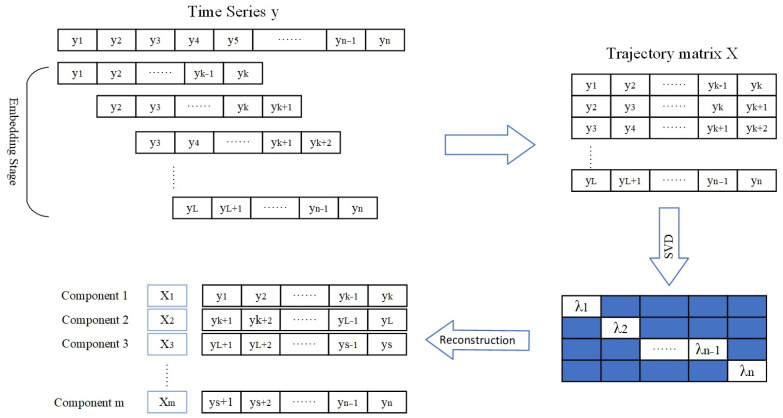
Flow chart of SSA.

SSA theory was proposed in the second half of the 20th century, and it is a rapidly developing method of time series analysis [28]. The SSA algorithm comprises two primary stages. The initial phase, termed decomposition, involves the transformation of a time series into a trajectory matrix, often referred to as a Hankel matrix. Subsequently, singular value decomposition (SVD) is employed on the trajectory matrix to affect a decomposition into elementary rank-one matrix components. The subsequent stage, termed reconstruction, ingeniously organizes matrix components into groups and reverts the grouped matrix decomposition to the decomposition of the original object via a process known as diagonal averaging. Figure 1 shows the process.

In the first stage, the trajectory matrix is formed by taking an equal length sequence from the input time series *x*, where the length of the subsequence is determined by the window length *L*, and it is usual to take $L<\frac{N}{2}$. The partitioned trajectory matrix is shown below.

$$X=\left[X_{1},X_{2},\cdots ,X_{K}\right]={\left(x_{ij}\right)}_{i,j=1}^{L,K}=\left[\begin{array}{ccccc} y_{1} & y_{2} & y_{3} & \cdots & y_{K}\\ y_{2} & y_{3} & y_{4} & \cdots & y_{K+1}\\ y_{3} & y_{4} & y_{5} & \cdots & y_{K+2}\\ \vdots & \vdots & \vdots & \ddots & \vdots \\ y_{L} & y_{L+1} & y_{L+2} & \ldots & y_{N} \end{array}\right]$$

Then, the singular value decomposition of *X* is performed using Equation (1) to obtain singular values.

(1) $$X=U\Sigma V^{T}$$

Set $S=XX^{T}$, and the eigenvalues of S are denoted by $\lambda_{1},\lambda_{2},\cdots ,\lambda_{L}$. In Equation (1), where $U\in R^{L\times L}$, $\Sigma \in R^{L\times K}$, $V\in R^{K\times K}$, Σ is the singular value matrix, which is a diagonal matrix, U and V are left and right singular vector matrices, respectively, and they are composed of the eigenvectors of $XX^{T}$ and $X^{T}X$, respectively.

Equation (1) can also be expressed as vectors:

(2) $$\begin{array}{ll} X & =\sum_{i=1}^{r}\sigma_{i}u_{i}v_{i}^{T}\\ & =X_{1}+X_{2}+\cdots +X_{r} \end{array}$$

where *r* is the rank of the matrix *X*, which is also the number of non-zero singular values, σ is the singular value, sorted in descending order in Σ. Therefore, submatrices can be expressed as $X_{i}=\sqrt{\lambda_{i}}U_{i}V_{i}^{T}$.

The second stage, or grouping part, starts with the eigentriple grouping, i.e., the collection $\left(\sqrt{\lambda_{i}},U_{i},V_{i}\right)$, aimed at dividing *X* into *m* linearly independent submatrices. The procedure is as follows:

Firstly, the index set $\left\{1,2,\cdots ,r\right\}$ is divided into m disjoint subsets $I_{1},I_{2},\cdots ,I_{m}$.

Let $I=\left\{i_{1},i_{2},\cdots ,i_{p}\right\}$ so that the resultant matrix *X_I_* corresponding to the group *I* is defined as $X_{I}=X_{i1}+X_{i2}+\cdots +X_{ip}$.

Then, resultant matrices can be calculated from $I_{1}$ to $I_{m}$.

Consequently, Equation (2) facilitates the decomposition, which can be expressed as follows:

(3) $$X=X_{I_{1}}+X_{I_{2}}+X_{I_{3}}+\cdots +X_{I_{m}}$$

The last step is the diagonal averaging, which starts with transforming $X_{I_{j}}$ into a new series of length *N*. Let Y be an $X_{I_{j}}$ matrix with elements $y_{ij},i,j\in \left[1,K\right]$, *N = L + K* − 1. If *L < K*, $y_{ij}^{*}=y_{ij}$ and $y_{ij}^{*}=y_{ji}$. By determining the diagonal averaging using Equation (4), the Y matrix is transferred into the new series $y_{1},y_{2},\cdots ,y_{N}$.

(4) $$y_{K}=\left\{\begin{array}{c} \frac{1}{k}\sum_{m=1}^{k}y_{m,k-m+1}^{*}\;\;1\leq k\leq L^{*}=\min\left\{L,K\right\}\\ \frac{1}{L^{*}}\sum_{m=1}^{L^{*}}y_{m,k-m+1}^{*}\;\;L^{*}\leq k\leq K^{*}=\max\left\{L,K\right\}\\ \frac{1}{N-k+1}\sum_{k-K^{*}+1}^{N-K^{*}+1}y_{m,k-m+1}^{*}\;\;K^{*}\leq k\leq N \end{array}\right.$$

After applying diagonal averaging to the resultant matrices, the reconstructed sequences are obtained: ${\tilde{X}}^{\left(k\right)}=({\tilde{X}}_{1}^{\left(k\right)},{\tilde{X}}_{2}^{\left(k\right)},\cdots ,{\tilde{X}}_{N}^{\left(k\right)})$. Thus, the initial series $x_{1},\cdots ,x_{N}$ can be represented by the sum of *m* reconstructed sequences.

(5) $$x_{n}=\sum_{k=1}^{m}{\tilde{x}}_{n}^{\left(k\right)}\;\;\left(n=1,2,\cdots ,N\right)$$

In light of the above, the effect of SSA is closely related to the number of decomposition layers and the window size. However, these parameters need to be tested constantly in the face of complex signals, which results in the failure of SSA to effectively separate the frequency components of complex signals.

Therefore, the clustering algorithm is introduced to provide the above parameters adaptively. When SVD is finished, K-means clustering is added to cluster the singular values, so that components with a similar frequency are effectively extracted.

As a widely used unsupervised learning algorithm, K-means can cluster unlabeled input data into different groups. Its principle is to divide the eigenmatrix X of a set of N samples into *K* clusters without intersection. The mean of all the data in a cluster is often called the “centroids” of the cluster.

In the K-means algorithm, the number of clusters, K, is a hyperparameter that must be specified by the user. The core task of K-means is to find K optimal centroids and classify the data closest to these centroids to the clusters represented by these centroids. In general, after the hyperparameter K is set, K centroids are randomly initialized; in practical application, K points in the sample are usually selected as the centroids for initialization. Equation (6) is used to consider the distance between the sample $x_{i}$ and the centroid $\mu_{k}$.

(6) $$d_{x_{i}\mu_{k}}={\left\|x_{i}-\mu_{k}\right\|}^{2}$$

Then it is necessary to find the value of *k* that minimizes $d_{x_{i}\mu_{k}}$, which also determines the centroids of the clusters to which this sample $x_{i}$ belongs. Subsequently, the clustering process can be finalized by computing the distance between each sample and its respective centroid, followed by selecting the sample with the minimum distance, which can be expressed by Equation (7).

(7) $$J(c_{1},c_{2},\ldots ,c_{m},\mu_{1},\mu_{2},\ldots ,\mu_{k})=\frac{1}{m}{\sum_{i=1}^{m}\left\|x_{i}-\mu_{c_{i}}\right\|}^{2}$$

where $c_{m}$ represents the subscript *k* of the centroid of the cluster to which the *m*-th sample belongs.

By adjusting different values of k, different centroids are obtained, and finally, the value of *J* in Equation (7) is minimized, thus completing the clustering process.

The flow chart of SSA combined with the K-means clustering algorithm is shown in Algorithm 1. In this work, the hyperparameter k of K-means clustering was obtained from a neural network described in the next part, the random state was set to 10, and other hyperparameters were set to default values.
**Algorithm 1.** Process of SSA combined with K-means clustering algorithm.**Input: Pending sequence *Y*(*t*)**1: ***Start***: Set window length *L*, if $L>\frac{N}{2}$, $L=\frac{N}{2}-1$, determine the value of clustering *K*; 2: Embed *Y*(*t*) into trajectory matrix *X*; 3: Perform singular value decomposition using Equation (1), and sort the singular values in descending order; 4: Perform K-means clustering, choose *K* singular values $\mu_{1},\mu_{2},\ldots ,\mu_{k}$ as clustering centroids; 5:  ***When*** new centroid is different from the original one; 6:    ***for*** *i* = 1 to *m*; 7:      Calculate the distance between the *i*th sample and the centroids according to Equation (6), and take the centroid with the smallest *d_xiμi_* and denoted as c*_i_*; 8:    ***end***; 9:    ***for*** *i* = 1 to *K*; 10:         Calculate the mean of the coordinate of all sample points belonging to the current centroid, and take the mean value as the new centroid. 11:  ***end***; 12: ***end***; 13: Screen for valid classifications based on correlations, selecting clusters with correlations greater than 0.99; 14: Filter the eigentriples corresponding to the singular values of each clustered cluster based on indexing; 15: Reconstruct the signal components with different frequencies according to Equation (4); 16: ***end***.**Output**: **Reconstructed signals**

### 2.2. Deep Residual Neural Networks

In order to extract meaningful components from the original signal, the K-means clustering algorithm is employed within the decomposition phase of SSA. However, determining the appropriate value of *K* is often challenging in practical applications. Thus, a deep residual neural network (Deep Res-Net) was applied to enhance performance, which is basically a convolutional neural network (CNN). In this work, it was optimized using residual subnets with soft thresholds.

**Figure 2:**
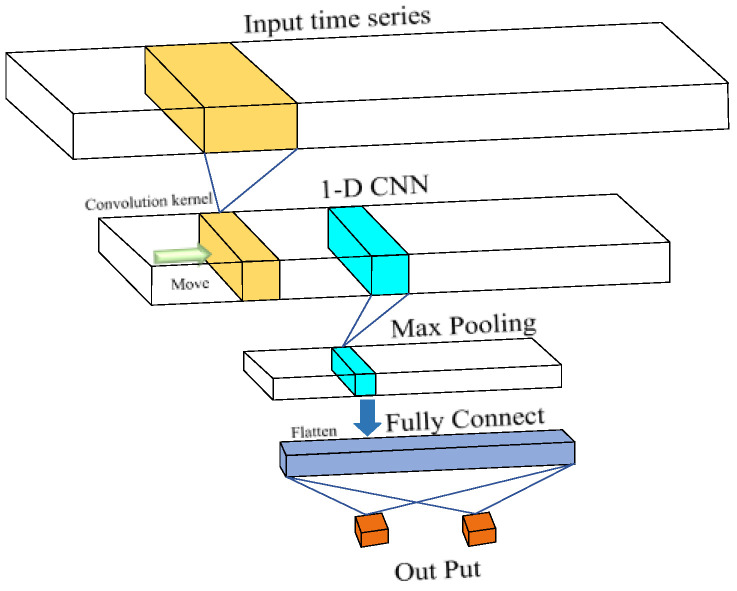
Illustration of basic CNN.

CNN has a great status in computer vision, but it can also be applied in time series processing, i.e., one-dimensional CNN (1-D CNN). The difference between 1-D CNN and 2-D CNN is the dimension of the convolution kernel. While 2D-CNNs have achieved remarkable recognition accuracy across various applications, they often necessitate the transformation of 1D data into 2D formats and typically require extensive datasets for training to mitigate the risk of overfitting. In contrast, 1D-CNNs offer a more compact architecture, enabling effective training on limited datasets of 1D signals. Furthermore, 1D-CNNs can be directly applied to raw signals without the need for extensive pre-processing or post-processing [29,30,31]. Given these advantages, 1D-CNNs were selected in this work to achieve an optimal balance between computational efficiency and accuracy. Figure 2 shows the basic 1-D CNN procedure.

In this work, ordinary CNN was optimized to achieve a better performance in 1-D signal recognition. The convolutional layer stands as the pivotal component distinguishing a CNN from traditional fully connected (FC) neural networks in machine learning architectures, and it also greatly reduces the number of trained parameters. This is accomplished by employing convolution instead of matrix multiplication, where the convolution kernels can possess fewer parameters compared to the transformation matrix in the FC layer. The relationship between the input and the convolution kernel can be expressed as follows:

(8) $$y_{j}=\sum_{i\in M_{j}}x_{i}*k_{ij}+b_{j}$$

where *x_i_* is the channel number of the input feature map, *y_j_* is the *j*-th channel of the output feature map, *k* is the convolutional kernel, *b* is the bias, and *M_j_* is a collection of channels that are used for calculating the *j*-th channel of the output feature map [32].

In the training process of CNN, theoretically, the model achieves better results as the number of network layers increases, which leads to a problem: vanishing or exploding gradients [33]. This problem has been usually addressed by normalized initialization and adding intermediate normalization layers [34,35]. Another approach to address this issue is through the utilization of deep residual networks. These networks represent an appealing variant of CNNs, leveraging identity shortcuts to alleviate the challenges associated with parameter optimization [36].

Research shows that the accuracy of CNN improves as the net gets deeper, but it faces the problem of exploding or vanishing gradients and degradation [37]. To improve the performance of CNN, residual blocks and soft thresholds are applied in this work. Residual blocks create a shortcut from input to output, allowing layers with poor performance to be skipped.

Compared to designing filters manually, using a gradient descent algorithm enables the net learn itself; therefore, combining soft thresholds with deep learning can be a good way to eliminate noise-related information and construct highly discriminative features [38]. The soft threshold function can be expressed as follows:

(9) $$y=soft(x,\tau )=\left\{\begin{array}{cc} x+\tau & x\leq \tau \\ 0 & \left|x\right|\leq \tau \\ x-\tau & x\geq \tau \end{array}\right.$$

where *x* is the input feature, *τ* is the threshold, which is a positive parameter, and the output feature is denoted by *y*.

**Figure 3:**
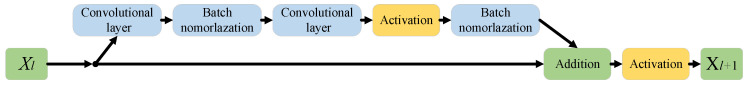
Basic principle of the residual block.

The basic principle of the residual block is shown in Figure 3, and a residual block can be represented as follows:

(10) $$x_{l+1}=f(x_{l})+F(x_{l},W_{l})$$

In Equation (10), $f(x_{l})$ is the direct mapping part, $F(x_{l},W_{l})$ is the residual part, corresponding to the upper and lower parts of the page, respectively.

**Figure 4:**
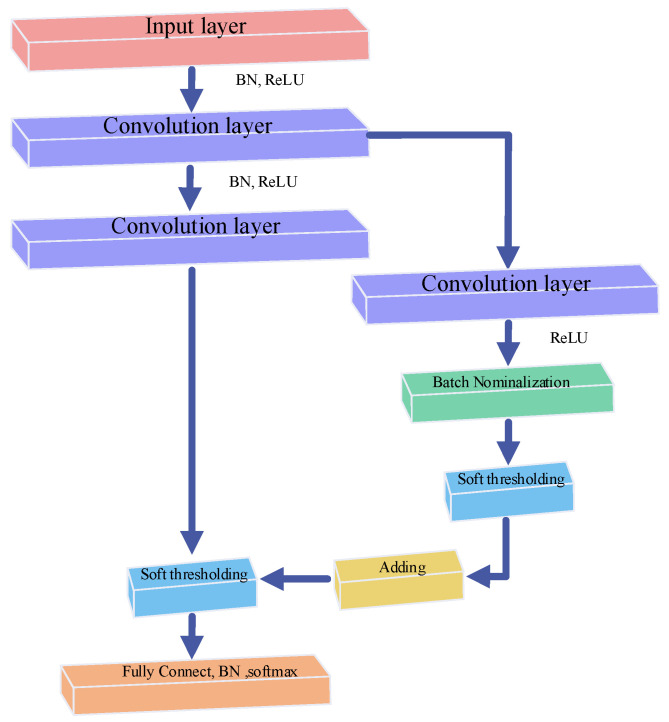
Diagram of Deep Res-Net for 1-D signal recognition.

Therefore, the whole flowchart showing the residual improved CNN is shown in Figure 4. The overall flow of ASSA is shown in Algorithm 2.
**Algorithm 2.** Overall workflow of the ASSA algorithm.**Input: Measured data sequence *Y*(*t*)**1: ***Start***: Deep Res-Net recognition process of *Y*(*t*); 2: Deep Res-Net outputs noise signal category *K* and target signal category *T*, respectively; 3: Set window length *L*, if $L>\frac{N}{2}$$,\;L=\frac{N}{2}-1$; 4: Embed *Y*(*t*) into trajectory matrix *X*; 5: Perform singular value decomposition using Equation (1), and sort the singular values in descending order; 6: Perform K-means clustering, choose *K + T* singular values $\mu_{1},\mu_{2},\ldots ,\mu_{k+T}$ as clustering centroids; 7: Obtain the clusters $C_{1},C_{2},\cdots ,C_{K+T}$, calculate the mean *C_i_* of *i*-th cluster $C_{i,mean}=\sum_{j=1}^{len(C_{i})}\frac{c_{ij}}{len(C_{i})}$, where *c_ij_* is the *j*-th element of *C_i_*, and sort in ascending order; 8: Set threshold *Ts*; 9: ***if*** $C_{i,mean}<Ts$; 10:    ***for*** *i*, *j* < *K + T*; 11:        ***if*** i≠j 12:            Correlation matrix $Cor\_m\left[i,j\right]=\left|1-\frac{C_{i,mean}}{C_{j,mean}}\right|$; 13:        ***end*** 14:    Valid cluster *C_v_* = *where* (*Cor_m* < 0.01) 15:    ***end*** 16: ***end*** 17: Filter the eigentriples corresponding to the singular values of each clustered cluster based on *C_v_*; 18: Reconstruct the signal components with different frequencies, according to Equation (4); 19: ***end***.**Output**: **Reconstructed signals**

## 3. Experiments and Result Analysis

To validate the effectiveness of the proposed algorithm, simulation experiments and practical experiments were carried out. For simulation experiments, a complex signal with different frequency components was constructed, and measured MT data were applied in practical experiments. The algorithm execution platform utilized in this experiment was: 12th Gen Intel(R) Core (TM) i9-12950HX (2.30 GHz), 3070Ti 8 GB GPU, 32 GB RAM, Windows 11 ×64 operation system. Software platform: Python 3.9.

### 3.1. Experiments of Frequency Resolution Performance

**Figure 5:**
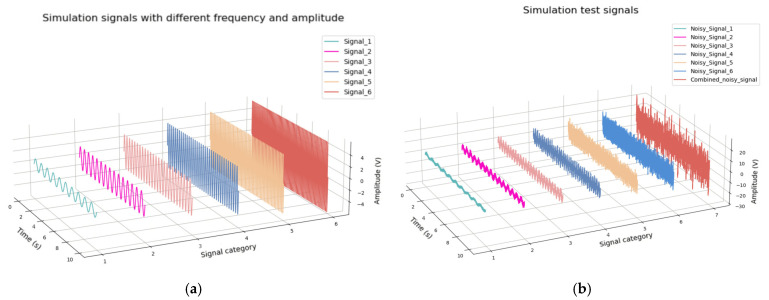
Simulation signals. (**a**) Original signals; (**b**) Simulation signals with background noise and combined signals.

**Figure 6:**
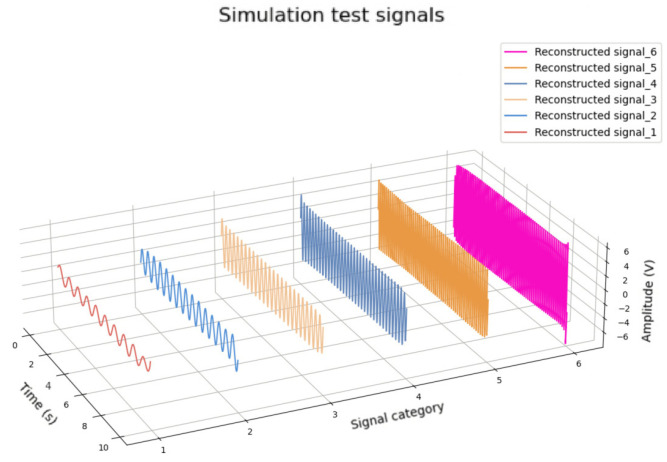
Decomposition results of sinusoidal signals with different frequencies and amplitudes.

In order to verify the ability of the proposed algorithm to recognize and decompose multiple frequency superposition signals, a complex signal with different frequency components was constructed. The simulation signal *s_1_* consists of sinusoidal signals with frequencies of 10, 15, 25, 35, 55, 65 Hz and amplitudes from one to six, respectively. Gaussian noise was also added to ensure that the simulation signals had a signal-to-noise ratio (SNR) of 3 dB. The simulation signals, simulation signals with Gaussian noise and combined signals are shown in Figure 5a,b, respectively. The decomposition result is shown in Figure 6. The root mean square error (RMSE) was used for quantitative analysis, as shown in Table 1.

From Figure 6, it can be seen that most of these signals were well reconstructed, with slight distortion in the range of about 100 sampling points at the beginning and end of the signal. It can also be seen that the amplitudes of the restored signals have a slight attenuation, but the overall levels are still maintained, and the higher the signal frequency, the more stable the reconstructed amplitude.

Through this experiment, it has been proven that the proposed method can effectively identify signals with close frequencies, and the signals are well reconstructed.

### 3.2. Experiments of Target Signal Recognition

The experimental network architecture comprises five residual subnetworks, each integrating two convolutional layers with ReLu and Sigmoid activation functions. The network was initiated with a primary convolutional layer, followed by five additional convolutional layers dedicated to channel parameter optimization. Through systematic hyperparameter tuning, the network achieves an optimal performance under the following configuration: Cross-entropy serves as the loss function, filter dimensions were progressively scaled from 16 to 256, a kernel of size 3 was implemented with He-normal initialization, the *l2* regularization coefficient was set to 10^−4^, and the learning rate was maintained at 0.001. This configuration demonstrated superior performance in temporal efficiency and model precision during the following experiments.

In the actual application scenario, the signal measured by the sensor was not an ideal sinusoidal signal, which is usually a complex composite signal with more frequency components. As mentioned above, MT observation data are susceptible to human interferences, and it is necessary to carry out simulation experiments of the corresponding interferences.

Therefore, a complex non-stationary signal *x* was constructed to verify the efficiency of the proposed method. The sawtooth function and square functions were used to generate large-scale triangular and square waves, and a single-sided attenuation pulse signal was used to simulate vibration interference. Based on different combinations of the above signals, different types of interference can be simulated so as to build a general interference database.

The simulation signal *x* has a sampling rate of 1200 Hz and sampling time of 10 s, *x = s + ns*, where *s* is a non-stationary signal, and *ns* is Gaussian background noise with a SNR of 3. For comprehensive analysis and discussion, the following four typical target signals were selected for comparative experiments:

$$\begin{array}{l} \text{Tringle}:s_{1}=sawtooth(2*\pi *50(t-1))\\ \text{Oscillating attenuation}:s_{2}=\exp(-10*(t-1)*\cos(2*\pi *50*(t-1))\\ \text{Pulstran}:\;s_{3}=pulstran(t-0.25,d,‘gauspuls’,10,0.5)\\ \text{Daul-frequency}:\;s_{4}=\exp(-10*(\left|y-7\right|))*\sin(2*\pi *50*(7-t)+\frac{\pi }{2}) \end{array}$$

**Figure 7:**
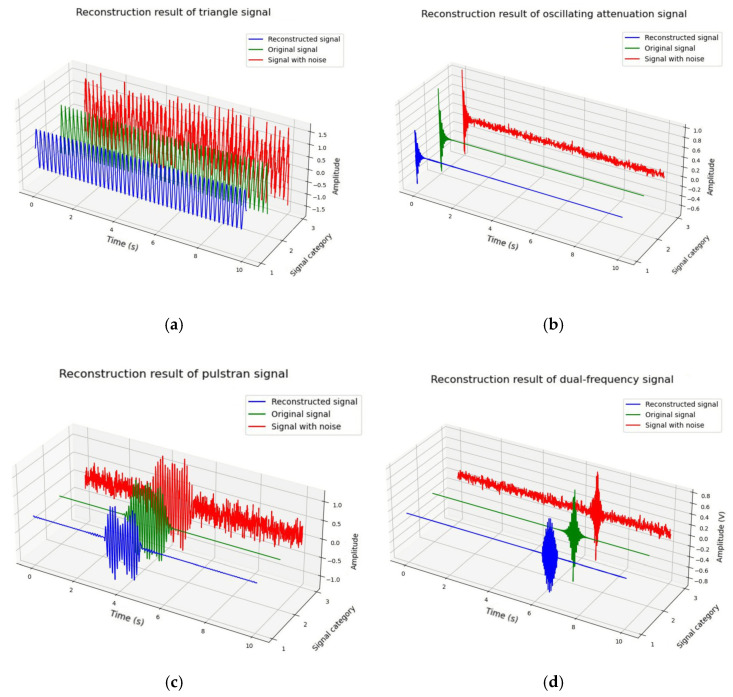
Decomposition and reconstruction results of simulated typical interference noise. (**a**) Comparison of triangle wave signal before and after reconstruction; (**b**) Comparison of oscillating attenuation signal before and after reconstruction; (**c**) Comparison of pulstran signal before and after reconstruction; (**d**) Comparison of dual-frequency signal before and after reconstruction.

During the experiments, all target signals were set as training data for the Deep Res-Net; the trained network was then applied to provide parameters for clusters of singular values. The recognition accuracy is up to 100%, which greatly enhances the accuracy of the K-means clustering algorithm and reduces the debugging time. The results are shown in Figure 7.

Figure 7a shows the reconstructed tringle wave signal. It can be seen that the reconstructed signal waveform is well preserved, with a slight attenuation in amplitude, and the signal amplitude rises after the 800th sampling point. Figure 7b shows the reconstructed oscillating attenuation signal, which has an attenuation of 0.4 amplitude at the initial time, but the overall target signal is well extracted. Figure 7c shows the restricted pulstran signal. The portion of the signal with an amplitude greater than 0 has a good extraction effect, while the portion of the signal with an amplitude less than 0 has a slight distortion, which is not smooth enough at the 800th sampling point. Figure 7d shows the reconstructed dual-frequency signal. The overall signal waveform is well extracted, and the background noise at the portion of the signal with an amplitude of 0 was not removed completely.

**Figure 8:**
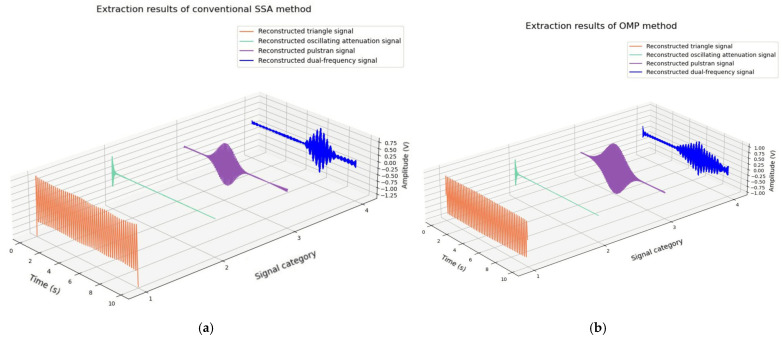
Comparison of simulation experiment results. (**a**) Simulation signal extraction results of SSA with manually adjusted parameters; (**b**) Simulation signal extraction results of OMP method.

In order to better demonstrate the effectiveness of the proposed method, the ASSA and RMSE methods were used to evaluate the extraction effect of the target signal. Conventional SSA with manually adjusted parameters and the orthogonal matching pursuit (OMP) algorithm were included for comparison, and the results are shown in Figure 8 and Table 1.

Figure 8 illustrates the performance of the SSA with manually adjusted parameters and OMP algorithms in extracting target signals, revealing varying degrees of distortion. For the triangular wave signal in Figure 8a, outliers are evident at the beginning and end, and the overall signal amplitude is unstable. While the general profile of the pulstran signal was reconstructed, it remains insufficiently accurate, with noticeable noise at the end. The dual-frequency signal was partially restored, but harmonic interference persists, and some target signal frequencies were not preserved. In Figure 8b, the triangle wave and oscillation attenuation signals are effectively reconstructed. However, the pulstran and dual-frequency signals exhibit significant distortion, with noise reduction remaining inadequate.

From Table 2, it can be seen that compared with conventional SSA and the OMP sparse signal extraction method, the proposed ASSA method demonstrates superior performance in recognizing and extracting typical interference signals. SSA, however, requires extensive manual parameter adjustments, often yielding suboptimal results with unquantifiable time expenditure. Without taking into account the debugging time, the processing speed of SSA is similar to that of ASSA. OMP suffers from the complexities of constructing over-complete dictionaries, leading to increased computational time and high hardware requirements. The ASSA method effectively addresses these limitations, offering a more efficient and robust solution.

### 3.3. Measured Data Experiments

**Figure 9:**
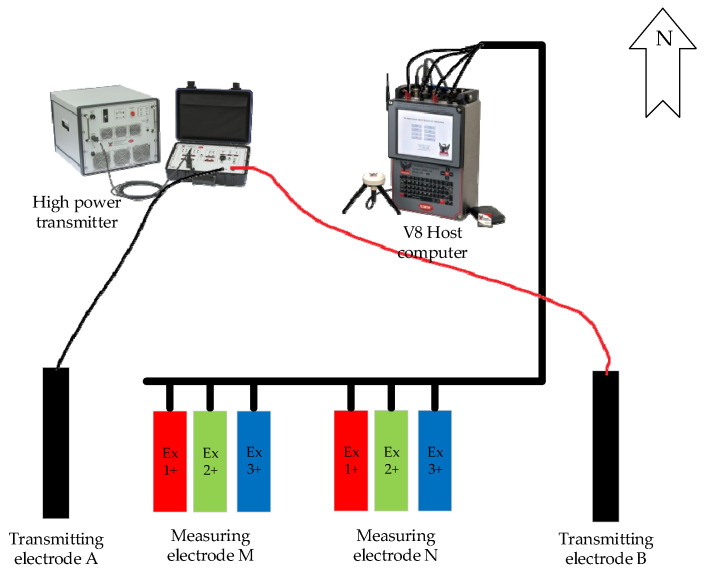
Sensing system schematic of the V8 MT system.

In order to verify the effect of the proposed method in practical application, experiments were carried out using measured data from the V8 MT system, developed by the Phoenix Corporation of Canada. The sensing system is shown in Figure 9.

In the practical application of the MT method, because the shape of the target signal was difficult to know in advance, and because there were complex noise interferences in the measurement process, the target signal could not be extracted effectively.

**Figure 10:**
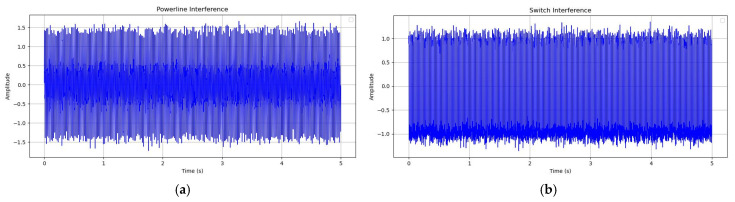

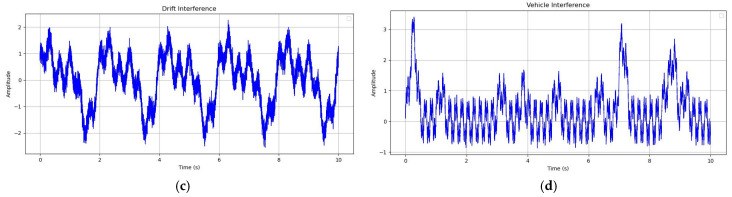
Time domain waveform of typical noise in the general noise database. (**a**) Powerline interference; (**b**) Switch interference; (**c**) Drift current interference; (**d**) Vehicle interference.

Before the experiment, additional work was carried out to collect and summarize the interference signals of the measurement area, including power frequency interference, stray current, electronic switch, motor noise, vehicle interference, etc., and the noise signal database was built during the simulation. Some typical interference signals are shown in Figure 10. From Figure 10, it can be seen that power line interference, usually from the power system, has a typical performance of 50 Hz sine wave and its harmonics, with the main energy concentrated in the middle of the signal. The main energy of the switch interference signal is concentrated in the bottom. The drift current interference signal is a kind of low-frequency, random or periodic interference signal and vehicle interference is the vibration and noise caused by engines, motors or tires, which manifests as the superposition of multiple low-frequency signals and some random transient pulses.

The Deep Res-Net was trained as follows: A simulation program generated 1000 noise signals, each with a sampling rate of 1200 Hz and a duration of 10 s. Because the amplitude of interference noise hardly exceeds 10 in real application, its amplitude starts at 0 and increases in 0.01 steps. These noise signals were then superimposed onto the target signal without noise and were finally used to establish a training dataset with dimensions of 1.2 × 10^3^ by 5000. The test set was similarly constructed by superimposing a variety of noise types onto a denoised target signal. Given that the sampling rate of the measurement equipment used in this experiment was also 1200 Hz, the measurement signals were segmented into 10 s intervals to maintain data size consistency. Each segment was processed individually to ensure both the proper functioning of the algorithm and the similarity between the training data and the measurement data.

Then, the noise signal database was employed in ASSA to eliminate interferences in the MT method so as to extract the target signal completely. In addition, the historical measurement data were also incorporated into the deep Res-Net training, which was used to identify the target signal type after the noise interference was eliminated to provide suitable parameters for SSA.

Typical interferences in the MT methods included power frequency interference, stray current, electronic switch, motor noise, vehicle interference, etc.

**Figure 11:**
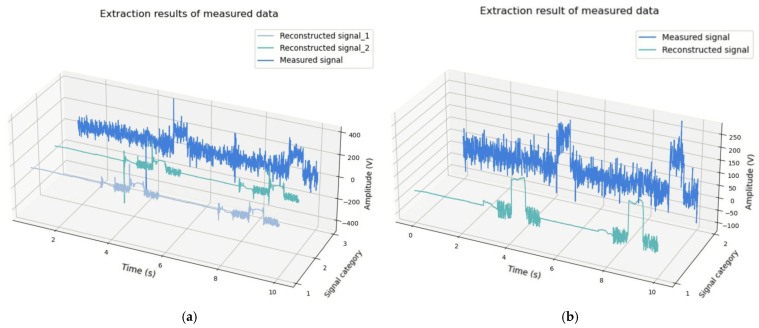
Signal recognition and extraction results of measured MT data. (**a**) First measurement extraction results; (**b**) Second measurement extraction results.

The test results are shown in Figure 11.

Figure 11 demonstrates that the original observed signal was significantly impacted by interference, leading to substantial disturbances. In contrast, the signal extracted using ASSA exhibits no distortion, achieves an exceptional disturbance elimination effect, and delivers a high signal-to-noise ratio (SNR). These improvements render the extracted signals highly suitable for subsequent inversion calculations.

In Figure 11a, the blue line represents the signal obtained from the first measurement, while the light blue and green lines correspond to the two useful signals extracted by ASSA, with root mean square errors (RMSE) of 0.2047 and 0.2902 and SNRs of 12 dB and 11 dB, respectively. Similarly, in Figure 11b, the blue line represents the second MT observation signal, and the green line indicates the target signal extracted by ASSA, which achieved an RMSE of 0.2962 and an SNR of 13 dB. Details are provided in Table 3.

**Figure 12:**
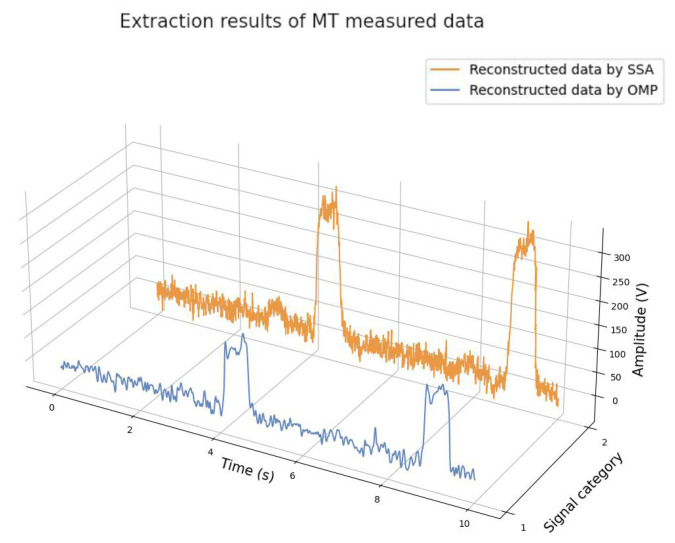
Second measurement extraction results by SSA and OMP.

For comparison, the conventional SSA and OMP algorithms were used to process the second measurement signal, and the results are shown in Figure 12 and Table 4:

As can be seen from Table 4, the processing time of traditional SSA is very close to that of ASSA, regardless of manual parameter adjustment. Although the extraction accuracy of the OMP algorithm was superior to that of ASSA, it consumed a lot of time due to the need to build a huge redundant dictionary.

**Figure 13:**
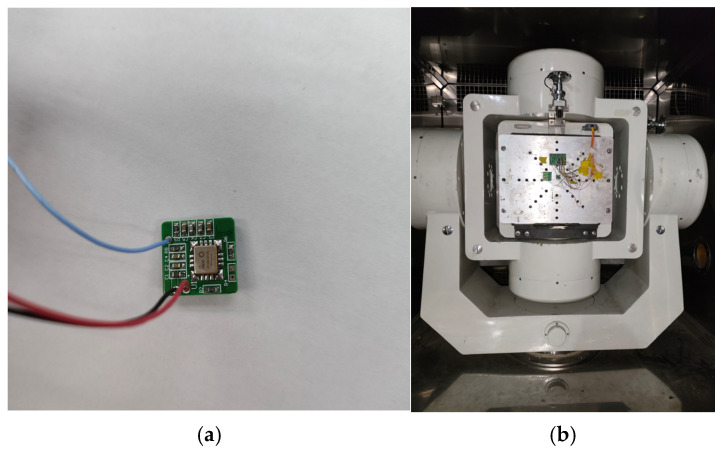
Pictures of CRM100 gyroscope and rotating platform. (**a**) CRM100 gyroscope; (**b**) Rotating platform.

In order to verify the generalization ability of the algorithm, another measured data experiment was carried out. The conducted experiment focused on the measurement data processing of an MEMS gyroscope, specifically utilizing a CRM100 gyroscope for data acquisition on a rotating platform. The zero position of the CRM100 gyroscope is 1.65 after conversion. Pictures of the CRM100 gyroscope and rotating platform are shown in Figure 13a,b, respectively.

**Figure 14:**
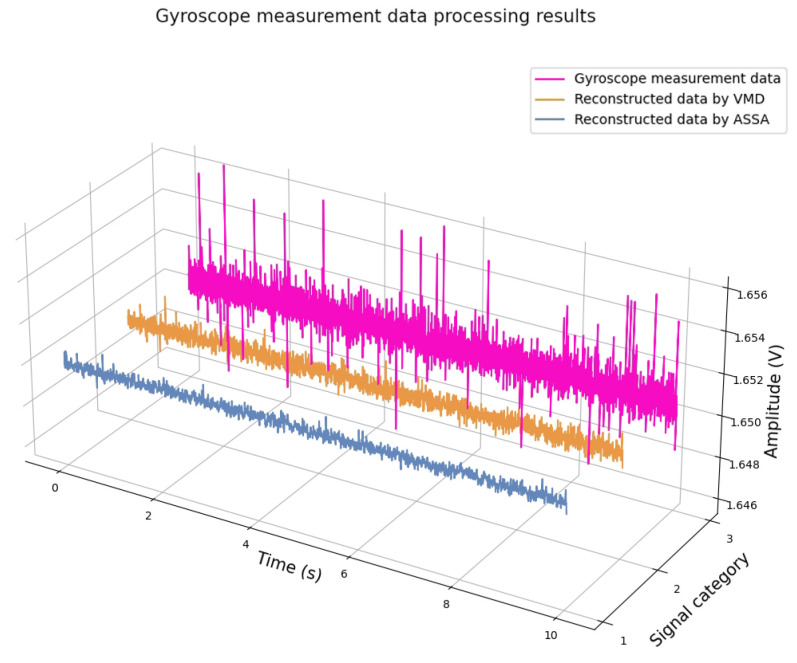
Processing results of gyroscope data.

Unlike MT data, the gyroscope’s noise is predominantly characterized by random walk noise, which is primarily manifested as Gaussian noise and weak current noise, mainly represented as weak damped noise. The model training process follows a similar methodology to that employed for MT data processing. Given the selected instrument’s sampling rate of 1 kHz, a noise dataset was constructed with a sampling rate of 1 kHz, a sampling duration of 10 s, and an amplitude range from 0 to 1. This dataset was then integrated with effective signals to form the training and test sets. During data processing, the gyroscope data was segmented into 10 s frames and processed sequentially. The results are shown in Figure 14 and Table 5.

It can be seen from Table 5 that ASSA has better signal processing performance in a similar processing time. The experiment proved that the proposed algorithm has good generalization ability.

## 4. Conclusions

This paper has presented an innovative adaptive singular spectrum analysis (ASSA) approach, integrating K-means clustering, Deep Res-Net, and a signal reconstruction method based on correlation detection to enhance signal recognition and noise extraction performance. By addressing the limitations of traditional SSA, which relies on manually adjusted parameters, the proposed method achieves automatic parameter optimization and robust adaptability. The ASSA framework is particularly advantageous for handling complex noise in multi-signal environments, providing higher extraction accuracy and faster processing speeds.

Through experiments, the performance of ASSA based on K-means clustering and Deep Res-Net proposed in this paper was verified, proving the accuracy of multi-signal recognition when the frequency is similar, and the ability of this method to identify and extract complex noises was also verified through simulation experiments. Compared with traditional SSA with manually adjusted parameters and the sparse decomposition signal extraction method, ASSA has higher extraction accuracy and stronger adaptability.

The experimental results show that this method is effective in practical applications. Compared with conventional data processing methods, ASSA eliminates the need for manual parameter adjustment and adaptively determines the optimal singular spectral decomposition layers, resulting in higher extraction accuracy and faster extraction speed. Furthermore, another advantage of ASSA is its versatility, as it can be applied to sensor measurement systems susceptible to complex noise, such as vibration detection and attitude measurement in high-dynamic environments. It also integrates well with other data analysis programs for researchers to use.

## Figures and Tables

**Table 1:** RMSE and processing time of each target frequency signal extraction.

Signal	RMSE	Processing Time (s)
Reconstructed signal 1	3.64 × 10^−2^	2.81
Reconstructed signal 2	3.45 × 10^−2^	2.73
Reconstructed signal 3	4.60 × 10^−2^	2.72
Reconstructed signal 4	4.26 × 10^−2^	2.99
Reconstructed signal 5	1.84 × 10^−2^	2.73
Reconstructed signal 6	3.64 × 10^−2^	2.72

**Table 2:** RMSE of each target signal extraction.

Signal	RMSE			Processing Time (s)		
	ASSA	SSA	OMP	ASSA	SSA	OMP
Triangle wave	**0.1041**	0.1512	0.4262	24.86	**21.75**	1075.91
Oscillating attenuation	**0.0356**	0.0473	0.0475	23.53	**23.37**	1075.91
Pulstran	**0.1889**	0.2163	0.2565	24.62	**20.58**	1075.91
Dual-frequency	**0.1552**	0.2367	0.2743	23.81	**20.79**	1075.91

**Table 3:** Experimental results of measured data processed by ASSA.

Reconstructed Signal	RMSE	SNR (dB)	Processing Time (s)
Reconstructed signal 1 (First time)	0.2047	12	32.75
Reconstructed signal 2 (First time)	0.2902	11	32.68
Reconstructed signal (Second time)	0.2962	13	33.57

**Table 4:** Experimental results of measured data processed by SSA and OMP.

Algorithm	RMSE	Processing Time (s)
Conventional SSA	0.4906	29.36
OMP	0.1342	4347.62

**Table 5:** Experimental results of gyroscope measured data.

Algorithm	RMSE	Processing Time (s)
ASSA	**3.57 × 10^−4^**	4.2
VMD	4.02 × 10^−4^	**3.3**

## Data Availability

The raw data supporting the conclusions of this article will be made available by the authors on request.
